# A Review of Hearing Loss in Cleft Palate Patients

**DOI:** 10.1155/2012/548698

**Published:** 2012-02-12

**Authors:** Bilal Gani, A. J. Kinshuck, R. Sharma

**Affiliations:** ^1^University of Liverpool, Cedar House, Ashton Street, Liverpool L69 3GE, UK; ^2^Department of Otolaryngology, Alder Hey Children's NHS Foundation Trust, Eaton Road, West Derby, Liverpool L12 2AP, UK

## Abstract

*Background*. Cleft palate is associated with recurrent otitis media with effusion and hearing loss. This study analysed the way these patients' hearing is managed in Alder Hey Children's Hospital. *Method*. A retrospective audit was carried out on cleft palate patients in Alder Hey Children's Hospital. Audiology assessment and treatment options were reviewed. Comparisons were made between the use of ventilation tubes (VTs) and hearing aids (HAs). The types of cleft, types of hearing loss, and the management output of the audiology regions were also reviewed. *Results*. The audiology assessments of 254 patients were examined. The incidence of VT insertion in this group of patients was 18.9%. The hearing aid incidence rate was 10.1%. The VT-related complication rate was 25.5% and the HA related complication rate was 9.1%. *Conclusion*. The data demonstrates that both treatments are viable, and a new protocol which combines the short term benefit of VT insertion with the lower complication rate of HA is required.

## 1. Introduction

Persistence of fluid in the middle ear, with an intact tympanic membrane, for a continuous period of three months or more is categorized as otitis media with effusion (OME) [1]. When the fluid persists, treatment may be required to reduce the impact of the conductive deafness, which can have consequences regarding the child's language, educational and social development.

 Certain individuals, such as those with cleft palate, are more likely to develop OME. The association between two has been well documented since Alt described the presence of otorrhoea in a child with cleft palate in 1879 [2]. Paradise et al. deduced that middle ear disease probably develops in all cleft palate patients [3]. However, more recent studies have confirmed this figure to be around 90% [4–6].

 Initially, repair of the cleft palate does slightly improve the ventilatory function of the eustachian tube. However, only after the adolescent growth phase, which also improves tubal function, does the incidence of OME greatly decrease [7].

 The high incidence of OME in cleft palate patients led to the conclusion that prophylactic treatment with ventilation tubes (VTs) would solve the inevitable hearing loss and prevent the sequelae of OME including cholesteatoma formation, retraction pockets, ossicular fixation, and atelectasis [8]. Studies have confirmed that early intervention with VTs does provide an appreciable benefit with regards to short-term hearing, between 6–12 months [9–11]. This regimen usually requires VTs to be inserted in the first year of life, at the same time as cleft palate surgery to reduce further operations [9]. However, currently the use of prophylactic VTs is being heavily scrutinised due to the associated complications; perforations, otorrhea, eardrum atrophy, granulation tissue, and tympanosclerosis (which have been reported to be as high as 80% [8]). Other studies have revealed that children who undergo multiple VT insertions increase their risk of conductive hearing loss in the long term [12].

 Maw et al. showed that 50% of OME resolves spontaneously within 3 months and that 90% of middle ear effusions occurring following acute suppurative otitis media resolve spontaneously [13, 14]. In view of this and the VT complication rate, a more conservative approach to treatment has ensued. Hearing aids (HAs) are now offered as an alternative to VTs, and insertion of VTs should only be considered in light of careful otological and audiological assessment [15].

**Figure 1:**
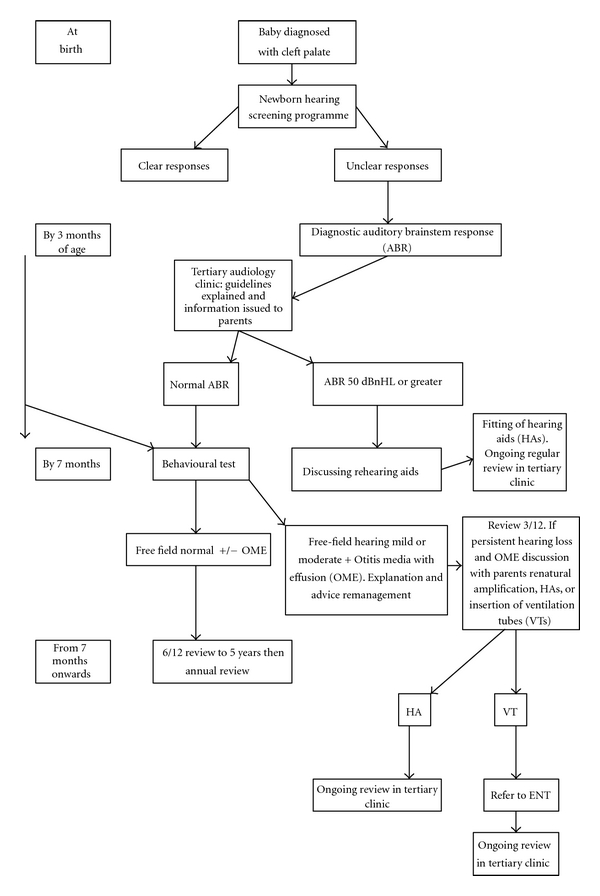
Audiological care pathway for children with cleft palate in the mersey region.

 The audiological assessment for cleft palate patients is continuous until the patient is discharged from the care of the cleft and audiology team. It begins with the newborn screening programme and regular audiology appointments follow, so that any hearing loss can be treated responsively. Even if the result from the tests indicates a clear response, careful prolonged assessment is required (Figure 1).

## 2. Methods

 The primary outcome of the study was to compare the hearing before and after the intervention(s) of HAs or VTs.

The secondary outcomes will examine a wide range of outcomes including:

- Distribution of cleft types.
- The types of syndromes, sequences, and anomalies found in the sample population.
- Complication rates of the treatments.
- Types of hearing losses within the sample.
- Whether the audiology centre predisposes to the treatment outcome (HA/VT).

Numerous variables were collected from the available data source for each patient (the appendix). The data was obtained from both primary sources (medical records) and secondary sources such as Meditech and audiology letters. This data was captured using a purpose-designed Microsoft Access database form. Chi-squared and Fisher's exact test, used to analyse the nominal data set using SPSS^©^. *P* values ≤ 0.05, were considered statistically significant.

 Audiological data was categorised according to the average degree of hearing across the 4 frequencies of 250 Hz, 500 Hz, 1 kHz, and 2 kHz obtained through primary or secondary analysis (the verdict of the audiologist). The categories for the degree of hearing loss ranged from normal <20 dB to profound >95 dB (derived from the British Association of Audiologists).

 As a result of the breadth of the data, the dataset encompasses periods where routine grommet insertion was used and more recently a selective procedure. In the latter period, the decision for VT insertion and HAs was made at the discretion of the audiologist and the ENT surgeon using the clinical triad of: (i) audiological evidence of hearing loss >20 dB, (ii) recurrent otitis media with persistent effusion ± anatomical abnormalities, and (iii) parental preference regarding hearing management.

 For any child to be included in the dataset, they must have had a cleft palate and be under the care of the cleft team. This excluded several patients with noncleft velopharyngeal insufficiency and or with cleft lip only. Patients who moved to outside the catchment area, discharged from the care of the cleft palate team, were deceased, and those who did not have aided hearing levels for HAs were excluded.

 Institutional ethical approval came from the Alder Hey Hospital audit department.

## 3. Results

 A retrospective audit was carried out on 254 consecutive children; under the care of the cleft palate team at Alder hey Children's hospital. The patients attended one of 14 audiology centres in north west England, North Wales, and the Isle of Man between the dates of 24/10/2009 to 08/03/2011.

 After the exclusion criteria, out of the 254 patients, 217 patients remained. Of which 63 were placed in the intervention group (HA(s)/VTs), and 154 were assigned to the watchful waiting group.

 The length of follow up in the intervention group varied from patient to patient. This was the time that had elapsed between their last pre-intervention audiology data record and their latest audiology data. This systematic approach yielded an average follow-up time of around 3 years.

### 3.1. Primary Outcome

 The primary outcome variable of hearing before and after the intervention was examined for each of the two intervention outcomes (Table 1). Only 40/63 patients were investigated, due to the timing of the intervention treatment (patients who had just received a new intervention had yet to have a postoperative assessment). Even so, this provided a value *P* = 0.47 indicating that in fact there was no significant association between hearing outcome and treatment, and by scrutinising the differences between the pre- and post values it can be deduced that both improve hearing outcomes.

### 3.2. Secondary Outcomes

#### 3.2.1. VT/HA Statistics and Complications

 The VT insertion rate was 18.9% (41/217), and the HA incidence rate was 10.1% (22/217). The total number of children who at some point had or were still wearing HAs was 38.1% (24/63). A similar analysis of VTs yielded 74.6% (47/63). The overlapping discretion was due to the 6 patients who had VTs first, followed by HA(s) and the 2 patient who had HA(s) first, followed by VTs (Table 2). 

 A large proportion of patients, 34.9% (22/63), had VTs inserted at the same time as cleft surgery. 

Of the 22.2% (14/63) patients that suffered from a complication 12 having VTs. Considering 47 patients had VTs at the time of the complications, the VT-related complication rate is 25.5% (12/47). The main complications derived VT insertions were tympanosclerosis (5 patients), perforation (5 patients), otalgia (2 patients), and retraction pocket(s) (1 patient). In total, this equates to 13 patients, the overlapping discrepancy is due to one of the patients having both perforation and tympanosclerosis.

 Similarly the HA related complication rate was 9.1% (2/22). The only recorded complication was noncompliant whereby the child would constantly remove the HA device. 

 A chi-square test of the current interventions and the complications would mask those complications that were due to previous VTs. When the data was reorganised to reflect when the complication was detected, a chi-square value of *P* < 0.05 was obtained indicating that the VTs were significantly associated with complications recorded. 

#### 3.2.2. Hearing Loss Types

 The predominant type of hearing loss in the intervention group was conductive, which affected 88.9% (56/63) of patients, whilst 7.9% (5/63) patients in the study had a mixed hearing loss and a minority of 3.2% (2/63) had a permanent sensorineural loss predominantly affecting their hearing. All sensorineural hearing loss patients received HAs and most conductive hearing loss patients received VTs (Table 3). 

#### 3.2.3. Type of Orofacial Clefts

The types of clefts are shown for the whole sample of 217 patients (Table 4) and the intervention group (Table 5). Both samples reflected a similar distribution of cleft types with a submucous cleft being the most rare and unilateral cleft lip and palate, hard palate only and hard and soft palate being the most populous. 

 The type of cleft was compared to the severity of preinterventional hearing loss. This returned a value of *P* > 0.05 indicating that the type of cleft does not significantly affect the severity of hearing loss that the patient will experience. 

#### 3.2.4. Syndromes/Sequences and Anomalies

A significant proportion of cleft palate patients had associated syndromes, sequences, and nonrandom associations. Just over a quarter, 27% (17/63), of patients that required interventions had a syndrome/sequence/association (Table 6). The Pierre Robin sequence was the modal condition accounting for 47% (8/17) of the syndromes/sequences/associations. 

#### 3.2.5. Audiology Regions and the Type of Interventions Implemented

 Since the introduction of the NICE guidelines in 2008, and with the majority of the patients being treated after the introduction of the guideline, it was intriguing to see whether there were discrepancies between the audiology regions and the type of treatments implemented. Across the board of 63 patients, there were 11 audiology centres involved (Table 7). After the current type of treatment was analysed against the region, a *P* *value* of 0.04 was obtained, which suggested that there was a significant link between the two.

## 4. Discussion 

 This study is comparable with those of Maheshwar et al., Phua et al., and Shaw et al. [2, 16, 17]. Comparisons with these studies must be made with caution due to the different clinical protocols and study designs implemented. However, it is universally accepted that children with cleft palate have a high incidence of middle ear disease, around 90%, before the age of 6. Up to 45% of these children will suffer from recurrent ear infections [18]. 

 Many studies have concentrated their efforts on one type of hearing management, namely, ventilation tubes. However, a more conservative approach has begun to influence OME management due to the ability of spontaneous resolution, and this is reflected with the introduction of hearing aids in this study. 

 The incidence of grommet insertion was 18.9% (41/217). This is lower than previous studies, for example, Shaw et al. 28% and Robertson et al. 26%, but it is comparative to that of Maheshwar et al. 17.1% [2, 17, 19]. These discrepancies may have arisen due to the outcomes of the different studies and the attitudes and clinical protocols of the time. 

 However, 46.8% (22/47) of the cleft palate patients who had or still have VT(s) underwent grommet insertions during a palatal repair or revision. This encompasses a range from 2002–2011 that reflects the earlier view of wanting to reduce the operative risk together with the present view that VTs should only be inserted at the time of cleft palate surgery if the child has glue ear, not as a prophylaxis. 

 Out of the 47 patients who had or still have VTs, 7 patients (14.9%), all born before 2007, required VT reinsertions suggesting that the ventilation tubes are in fact ineffective at preventing reinfection once shed. This study agrees with Merrick et al. (28%) assuming that the figures are relative to the change in clinical protocols [20]. 

 The HA incidence rate was 10.1% (22/217). Interestingly, 27.2% (6/22) of patients had previous VTs before being managed with HAs. This may be linked to the fact that patients who undergo a greater number of grommet insertions have a higher number of tympanic membrane deformities [16]. This is illustrated by 28.6% (2/7) patients who underwent repeated grommet insertions, developing perforations of the tympanic membrane. 

 Approximately 19.0% (12/63) patients who required an intervention developed visible or symptomatic complications upon audiological assessment by way of audiograms, tympanograms, and otoscopy. This is slightly higher but comparable to Maheshwar et al. (17.1%), but a lot lower than some aforementioned studies [8]. VTs were also found to be significantly linked to the complications. However, one has to bear in mind that a false negative picture may bias the results for two reasons. Firstly, because of the sample size used with regards to the intervention group and secondly, because of hearing aid complications such as compliance, wax build up, or ear infections not being fully documented as they may have been treated by the General Practitioner locally or are not recognised as a possible complication of the device, leading to a false negative picture. 

 Similarly with regards to the type of hearing loss and the treatment implemented one has to factor in things such as children accepting hearing aids and not pulling them out and parents' not wanting their children to have a visible hearing aid. This means that for a conductive or mixed hearing loss HAs or VTs can be used. Although for sensorineural hearing loss, HAs are solely used. 

 Due to the uncertainty of the optimum treatment plan, there are different preferences between the audiology centres. For example, in this dataset the audiology centre in Preston dispenses the highest number of hearing aids when compared to the Wirral and St Helen audiology centres which preferred VTs. This highlights the lack of uniformity in the treatment protocol. 

 The primary outcome measure of hearing outcome in relation to HAs and VTs indicated that there was no significant link between the two variables. According to this study, in terms of audiological improvement, both types of treatment are on par with each other. 

## 5. Conclusion 

 The results from this study show lower VT complication rates when compared to other similar studies. This may be linked to the greater implementation of HAs, which are equal to the VTs in terms of improving audiological outcome. On the whole, HAs are also better tolerated than VTs by the majority of patients, with noncompliance being the sole complication issue. 

 The disparity in the field is reflected by some audiology centres preferring either HAs or VTs, and this echoes the current knowledge based on this topic. However, it is worth considering that factors such as parental decisions, speech and language outcomes, and cost effectiveness will influence the treatment outcome meaning that complete uniformity with regards to a stringent treatment protocol will not be attainable. 

## Figures and Tables

**Table 1:** Comparing treatment outcomes by way of differences in pre- and post-intervention hearing.

Difference between preintervention and postintervention hearing versus hearing intervention				
		Hearing intervention		Total
		HA	VT	

Difference between preintervention and postintervention hearing	−1	0	2	2
	0	2	5	7
	1	8	12	20
	2	2	9	11

Total		12	28	40

**Table 2:** Treatments Instituted. VT→HA = ventilation tubes first followed by hearing aids. HA→VT = Hearing aids first followed by ventilation tubes.

Hearing interventions past and present		
	Frequency	Percent (%)
HA	16	25.4
VT	39	61.9
VT→HA	6	9.5
HA→VT	2	3.2

Total	63	100.0

**Table 3:** Comparing treatment outcomes by way of differences in pre- and post intervention hearing.

Hearing loss type versus hearing intervention				
		Hearing intervention		Total
		HA	VT	

Hearing loss type	Conductive	17	39	56
	Sensorineural	2	0	2
	Mixed	3	2	5

Total		22	41	63

**Table 4:** Distribution of cleft types in the sample. UCLP: unilateral cleft lip and palate; BCLP: bilateral cleft lip and palate.

Type of Cleft	Frequency	Percentage (%)
Soft palate	55	25.34
Hard palate and soft palate	61	28.11
UCLP	61	28.11
BCLP	33	15.21
Submucous	7	3.23

Total	217	100

**Table 5:** Distribution of cleft types in the intervention group. UCLP: unilateral cleft lip and palate; BCLP: bilateral cleft lip and palate.

Type of cleft		
	Frequency	Percent (%)
Soft palate	19	30.2
Hard and soft palate	15	23.8
UCLP	17	27.0
BCLP	11	17.5
Submucous cleft	1	1.6

Total	63	100.0

**Table 6:** Associated syndromes, associations and nonrandom anomalies. PR: Pierre Robin sequence.

Syndrome/sequence/association		
	Frequency	Percent (%)
No syndrome	46	73.0
PR	8	12.7
Charge	1	1.6
Crouzons	1	1.6
Digeorge	1	1.6
Goldenhar	1	1.6
Kabuki	2	3.2
Orofacial digital	1	1.6
Van der woude	1	1.6
Stickler and PR	1	1.6

Total	63	100.0

**Table 7:** Audiology centres and type of treatment outcomes.

Audiology centres versus hearing interventions				
		Hearing intervention		Total
		HA	VT	
	Alderhey	0	3	3
	Preston	13	6	19
	Southport	2	3	5
	Chester	0	1	1
	Crewe	1	3	4
Audiology centres	Wirral	1	6	7
	St Helen	1	8	9
	Isle of Man	0	1	1
	Wrexham	2	5	7
	Warrington	1	5	6
	Wigan	1	0	1

Total		22	41	63

**Table 8:** 

Variable	Explanation
Syndromes/sequences/associations	All anomalies associated with orofacial clefts were collected.
Preintervention hearing outcome	For those who received a management intervention, this would be the last available audiological assessment prior to the intervention.
Postintervention hearing outcome	This would be the most recent audiological assessment after the intervention.
Type of cleft	This was classified as cleft of soft palate, cleft of hard and soft palate, unilateral cleft lip and palate, bilateral cleft lip and palate and cleft lip only.
Complications	The complications recorded were those that occurred during or directly after the intervention.
Regions	This would be categorised according to one of the 14 centres in the locality.
Type of intervention	The expanded data set is HA, VT, HA + VT, HA→VT, VT→HA, and watchful waiting.
Type of hearing loss	One of sensorineural, mixed, and conductive.

## Appendix A

See Table 8.
